# Beyond adrenarche: practical tips for identifying virilization in young girls

**DOI:** 10.1093/pch/pxag049

**Published:** 2026-07-20

**Authors:** Sophie Lamoureux, Elizabeth A Cummings

**Affiliations:** NOSM University, Sudbury, Ontario, Canada; Dalhousie University, Halifax, Nova Scotia, Canada; Division of Paediatric Endocrinology, IWK Health, Halifax, Nova Scotia, Canada

## Introduction

Pubarche describes the development of pubic hair due to androgen-dependent maturation of pilosebaceous units, often accompanied by androgen-mediated features such as apocrine body odour, acne and axillary hair. It occurs as part of normal development or may present early as premature pubarche, defined as pubic hair before age 8 years in girls. Most cases reflect benign premature adrenarche; however, pubarche may also result from pathologic states such as congenital adrenal hyperplasia, androgen producing tumours or exogenous androgen exposure (1–3).

In contrast, virilization refers to pathologic male-pattern features (e.g., clitoromegaly, voice deepening, increased muscle mass), which are distinct from normal pubarche and are always abnormal. Accelerated linear growth, particularly deviation from a previously established growth trajectory (no longer tracking parallel to centile curves), is a common accompanying feature that should heighten concern (1–3). In the era of virtual care, an in-person assessment is essential when there is a question of pubertal changes to accurately assess Tanner stage and growth velocity. Rapid progression of androgenic features, including clitoromegaly or voice deepening, suggests pathologic androgen exposure requiring immediate investigation to prevent long-term sequelae. This article outlines practical tips to help paediatricians recognize red flags, interpret targeted investigations and identify exogenous androgen exposure.

## Case vignette

A 4-year-old girl presented with a 6-month history of pubic hair development and clitoromegaly. Growth acceleration was noted, with height increasing from the 50th to above the 97th percentile over 6 months.

Biochemical evaluation revealed markedly elevated testosterone (5.8 nmol/L). Dehydroepiandrosterone sulfate (DHEAS), androstenedione and 17-hydroxyprogesterone (17-OHP) were within normal limits for age, indicating no biochemical evidence of adrenal tumour or congenital adrenal hyperplasia due to 11- or 21-hydroxylase deficiency. Pelvic imaging was unremarkable. Bone age was mildly advanced.

Further history identified her father's use of a compounded 15% testosterone cream applied to his wrists as the presumed source of secondary exposure. When he transitioned to use of intramuscular testosterone, the child's testosterone levels rapidly normalized with some regression of virilization. Early recognition likely prevented complications such as secondary central precocious puberty and irreversible premature epiphyseal maturation, while allowing for partial regression of hyperandrogenic features.

## Practical tip #1

### Distinguish benign premature adrenarche from virilization

Premature adrenarche is usually a benign process characterized by slowly progressive pubic or axillary hair development and can be accompanied by mild acne or body odour but no breast development. Growth velocity is normal. In contrast, virilization is characterized by clitoromegaly, markedly accelerated linear growth, or rapidly progressive androgenic changes and is never benign (1–3).

Bone age may be advanced in up to one-third of children with benign premature adrenarche and should be interpreted in the context of the overall clinical picture (Table 1) (2,3).

**Table 1:** Key distinctions between benign adrenarche and pathologic virilization.

Feature	Benign adrenarche	Pathologic virilization
**ONSET**	Gradual	Rapid or abrupt
**GROWTH PATTERN**	Normal or mildly accelerated	Moderately to markedly accelerated
**CLITOROMEGALY**	Absent	Present
**BONE AGE**	Normal or mildly advanced*	Advanced (often disproportionate to linear growth)
**HORMONE PROFILE**	Mildly ↑ DHEAS only	↑ Testosterone; ↑ DHEAS and 17-OHP (adrenal source)
**TYPICAL SOURCE**	Adrenal maturation	Adrenal/ovarian tumour, congenital adrenal hyperplasia or exogenous androgen

^*^Bone age advancement may be seen in benign premature adrenarche and is often proportionate to linear growth (2).

A careful physical examination is essential, with attention to Tanner staging, genital anatomy and growth patterns. The presence of clitoromegaly should prompt further investigation and potential referral to paediatric endocrinology.

## Practical tip #2

### Always ask about exogenous androgen exposure, including compounded products

When endogenous causes have been excluded, exogenous androgen exposure must be considered. Household use of topical testosterone preparations, including compounded or “bioidentical” formulations, is an underrecognized cause of paediatric virilization (4,5).

Compounded testosterone products may contain supraphysiologic concentrations (containing up to 15% testosterone versus 1% to 1.62% in standard commercial testosterone gels) and have unpredictable absorption and prolonged transfer potential (4,5). Application to areas typically covered by clothing (e.g., upper outer arm/shoulder), thorough handwashing by the testosterone user following application and avoidance of skin-to-skin contact with the application site or contaminated materials (e.g., clothing, linens) are essential to reduce secondary exposure. Laundering clothing and linens belonging to the testosterone user separately may further reduce transfer.

Direct questioning about hormone-containing products (e.g., topical testosterone, anabolic supplements or hormone-replacement products) used by household members is essential. Identification of exposure should prompt immediate caregiver education and consideration of alternative formulations. Early intervention can halt progression of virilization and prevent long-term complications.

## Practical tip #3

### Use targeted biochemistry to localize the androgen source

Initial biochemical evaluation should include testosterone, DHEAS, androstenedione and 17-OHP. Testing should be guided by clinical findings and aimed at identifying the source of androgen excess (e.g., adrenal, ovarian or exogenous causes) (1,6). In benign premature adrenarche, DHEAS is minimally increased to early pubertal levels (2). It is important to remember that steroid hormone reference ranges vary significantly by assay and should be interpreted using age- and assay-specific norms, such as those available through the CALIPER database (7).

Significant elevation in androgens requires investigation for a virilizing ovarian or adrenal tumour or congenital adrenal hyperplasia, most often associated with elevated 17-OHP. Isolated elevated testosterone with normal DHEAS and 17-OHP should raise suspicion for ovarian pathology or exogenous androgen exposure (1,6). Bone age assessment may be advanced but may be less informative in recent onset androgen exposure. Table 1 summarizes key clinical and biochemical patterns that assist in diagnosis and guide imaging investigations.

## Summary of practical tips

- Virilization, including clitoromegaly, is always abnormal and distinct from benign premature adrenarche.
- Ask specifically about household use of hormone-containing products, including compounded testosterone creams, which carry higher transfer risk than standard commercial topical formulations.
- Initial biochemical testing can help localize the source of androgen excess and guide further evaluation.
- Early recognition and androgen source control can prevent long-term sequelae (such as secondary central precocious puberty and premature epiphyseal maturation) and may allow partial regression of hyperandrogenic features.
